# An Overview on the Field of Micro- and Nanotechnologies for Synthetic Peptide-Based Vaccines

**DOI:** 10.1155/2011/181646

**Published:** 2011-06-15

**Authors:** Aiala Salvador, Manoli Igartua, Rosa Maria Hernández, José Luis Pedraz

**Affiliations:** ^1^NanoBioCel Group, Laboratory of Pharmaceutics, School of Pharmacy, University of the Basque Country, 01006 Vitoria, Spain; ^2^Biomedical Research Networking Center in Bioengineering, Biomaterials and Nanomedicine (CIBER-BBN), 01006 Vitoria, Spain

## Abstract

The development of synthetic peptide-based vaccines has many advantages in comparison with vaccines based on live attenuated organisms, inactivated or killed organism, or toxins. Peptide-based vaccines cannot revert to a virulent form, allow a better conservation, and are produced more easily and safely. However, they generate a weaker immune response than other vaccines, and the inclusion of adjuvants and/or the use of vaccine delivery systems is almost always needed. Among vaccine delivery systems, micro- and nanoparticulated ones are attractive, because their particulate nature can increase cross-presentation of the peptide. In addition, they can be passively or actively targeted to antigen presenting cells. Furthermore, particulate adjuvants are able to directly activate innate immune system *in vivo*. Here, we summarize micro- and nanoparticulated vaccine delivery systems used in the field of synthetic peptide-based vaccines as well as strategies to increase their immunogenicity.

## 1. Introduction

In recent years, there has been an increase in the development of vaccination technology, but the ideal vaccine has not already been found. In general terms, there are some criteria which a vaccine must satisfy; it must be capable of eliciting the appropriate immune response, and it should be safe, stable, and reproducible. There are other issues such as cost, number of administrations, or immunization route which may also have to be taken into account [1]. Traditional vaccines have been developed using live attenuated organisms (such as BCG—Bacillus Calmette-Guerin, measles, mumps, rubella, and varicella), killed or inactivated whole organisms (e.g., influenza) or inactivated toxins (including diphtheria and tetanus) [2]. Live vaccines have the advantage of producing both humoral and cellular immune responses and often require only one boost. However, these vaccines are environmentally labile and require refrigeration, making difficult the delivery of these therapeutic agents, especially in the developing countries. Furthermore, the use of attenuated pathogens can revert to a more active form, a danger particularly acute in immune-compromised individuals [3]. Killed or inactivated organisms generate a weaker immune response and typically require multiple doses [4]. Hence, these types of vaccines generally require the addition of an adjuvant to be effective [5]. These disadvantages led to the development of subunit vaccines, including synthetic peptides as antigen, which consist of a specific part of the whole pathogen which has been demonstrated to stimulate an immune response. These vaccines are attractive, because they cannot revert to their virulent form and can be produced in bulk, safely and reproducibly. However, subunit vaccines have relatively low immunogenicity [6] which makes necessary the use of adjuvants and/or vaccine delivery systems. Besides, protein-based vaccines may be degraded by protease activity and have limited bioavailability, since they often cannot cross biological membranes [7, 8]. Finding the optimal combination between a given synthetic peptide and an adjuvant opens an unlimited clinical potential for these vaccines, because if adequate epitopes were identified for a certain disease, antigens could be synthesized on demand. For this reason, successful adjuvants need to be safe and well tolerated, simply produced and with inexpensive compounds, biodegradables, compatibles with many different antigens, and capable of function as a delivery system and immune potentiators [9]. Therefore, for licensing of new or newly formulated vaccines, nonclinical and clinical data regarding safety and efficacy are required, next to pharmaceutical quality data. These data are needed on the active ingredients, as well as the adjuvants and delivery systems, and their combination in the final product [10]. In this regard, there is only one guideline specifically dedicated to peptides, *Guidance for Industry for the Submission of Chemistry*, *Manufacturing*, *and Controls Information for Synthetic Peptide Substances*, published in 1994 [11], which stipulates the lot release specifications (sufficient to ensure the identity, purity, and strength of the peptide and demonstrate lot-to-lot consistency).

The need of eliciting both humoral and cellular immune responses has limited the efficacy against certain pathogens, such as malaria and HIV [3]. Activating the cytolytic immune response (CTL) is needed in the case of intracellular pathogens or tumors, and it is mediated by CD8 T cells, CD4 Th1 cells and natural killer T cells. Dendritic cells (DCs) have several innate features that make them ideal targets for vaccination purposes. They can capture antigens that enter the body and move to the T cell areas of lymphoid organs to find the right clones and start the immune response [10]. In peripheral tissues, DCs are found in an immature stage specialized in capturing foreign antigens. In response to microbes, DCs undergo a process of maturation into antigen-presenting cells (APCs). Meanwhile, they migrate from the periphery to the draining lymph nodes, where they present antigens to the T lymphocytes. DCs can present peptides to the T cells in the context of major histocompatibility complex (MHC) classes I and II molecules and also glycolipids and glycopeptides to T cells and NKT cells as well as polypeptides to B cells [12]. In order to achieve a CTL response, cytolytic cells must specifically recognize pathogen-derived antigens presented in MHC class I or in the CD1-lipid complex. Upon antigen recognition, immune cells release cytolytic agents that directly destroy infected cells and can induce inflammatory reactions which facilitate innate immune clearance and the development of some humoral response. 

**Figure 1:**
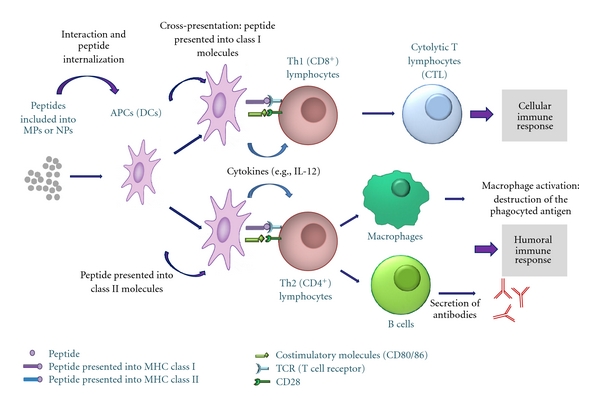
Schematic overview of the immune response developed after vaccination with micro- and nanoparticles entrapping antigenic peptides.

In order to generate CD8^+^ T cell immune responses cross-presentation have to occur, in which an exogenous antigen is presented into MHC I molecules in order to promote strong cytolytic and Th1 inflammatory bias [3]. Most protein-based vaccines do not develop cytolytic responses, because they are more readily processed into MHC class II molecules (which triggers humoral or antibody-dependent immune responses) [13]. For the development of a CTL response, antigens have to escape from the endosomal compartment into the cytosolic and endoplasmic reticular space, where the cross-presentation occurs [3]. Micro- and nanoparticle-based vaccine delivery systems can function as antigen carriers. Their particulate nature has some inherent ability to facilitate antigen cross-presentation [3], since they resemble pathogens particulate structure that looks like the biological situation. Particles *per se* are passively directed to the APCs and can increase the interaction between these cells and the antigen due to particles slow degradation [1]. Apart from the depot effect, particulate adjuvants can directly activate innate immunity *in vivo* [14]; that is, they work as immunoadjuvants. Thus, modification of these systems to directly target APCs may be a good approach for improving their efficacy. Therefore, micro- and nanoparticulated delivery systems can lead good opportunities in the development of synthetic peptide-based vaccines (Figure 1). 

When preparing micro- or nanodevices, there are some key formulation aspects such as chemical composition and manufacturing process, which affect the antigen loading capacity and release profile, product stability, efficacy, and safety [15]. For instance, the difference in size between micro- and nanoparticles may change the immune response achieved. The smaller the particle, the greater the proportion of drug located on its surface. This can lead to a substantial loss of payload or to a lower maximal drug loading for smaller particles [16], which finally may affect to the adjuvant activity. Moreover, the preparation process of micro- and nanoparticles can lead to stability problems due to the exposure to strong stress conditions (e.g., aqueous/organic interfaces, hydrophobic surfaces, and vigorous shaking) [17]. For this reason, peptide stability, once entrapped into the formulation, should be evaluated, since it is unlikely to develop a universal encapsulation approach appropriate to every peptide. For instance, in order to study the stability of the SPf66 peptide encapsulated into PLGA MPs, Carcaboso et al. [18] analyzed peptide integrity by polyacrylamide gel electrophoresis and showed no bands indicating partial degradation or aggregation of the protein.

Nowadays, there are no marketed vaccines composed of synthetic peptides. However, there are approved vaccines based on micro- and nanotechnologies. Alum is the most widely used adjuvant for human vaccines in the form of particulated aluminium salts (generally, Al(OH)_3_ and AlPO_4_) [19]. As shown in Table 1, it is used in various vaccines, such as the combined vaccine containing antigens against diphtheria, tetanus, and pertusiss [20] and against hepatitis B (Recombivax HB [21] or Engerix B [22, 23]). More recently, other particulate adjuvants have been licensed for human use. Emulsions like MF59 or AS03 are components of Fluad and Pandemrix, respectively [24, 25]. Other vaccines such as Epaxal [26] or Inflexal [27] include virosomes. Latest approved systems are composed of combination of adjuvants, such as AS04 (approved for human use in both Europe and USA), which comprises MPL (monophosphoril lipid A) and alum and is used into Fendrix [28] or AS04 combined with virus like particles (VLPs) used into Cervarix [29, 30] and Gardasil [31].

This paper summarizes micro- and nanoparticulated delivery systems used in the development of synthetic peptide-based vaccines. We also discuss various strategies for improving their efficacy in developing an appropriate immune response (Table 2).

## 2. Micro- and Nanoparticulated Systems for Synthetic Peptide Vaccine Development

### 2.1. Alum

Aluminium salts (generally, Al(OH)_3_ and AlPO_4_), often called alum, have been widely used in humans for more than 80 years, and, until recently, it has been the only adjuvant approved for human use in the USA [32]. Currently, there are many vaccines containing alum, such as Recombivax HB or Engerix B. Alum adjuvancity is associated with enhanced antibody responses [19]. It has been shown that after OVA-alum administration Th2 effector response is generated, as T helper cells produced IL-4, IL-5, and IL-10 but little IFN-*γ* [33]. In addition, Li et al. demonstrated that alum enhances the production of IL-10, a Th2 cytokine, and inhibits that of IP-10 (IFN-*γ*-inducible protein), a chemokine specific for Th1 cells [34]. It has been shown that alum induces rapid cell recruitment at the injection site. Kool et al. demonstrated that after an intraperitoneal injection of alum, a local production of chemoatractants like CCL2 and CXCL1 was triggered, as well as a recruitment of neutrophils, eosinophils, monocytes, and subsequently DCs. This study also revealed that following intraperitoneal or intramuscular administration of alum, recruited monocytes migrate to the draining lymph nodes and differenciate into inflammatory DCs capable of priming T cells [33].

**Figure 2:**
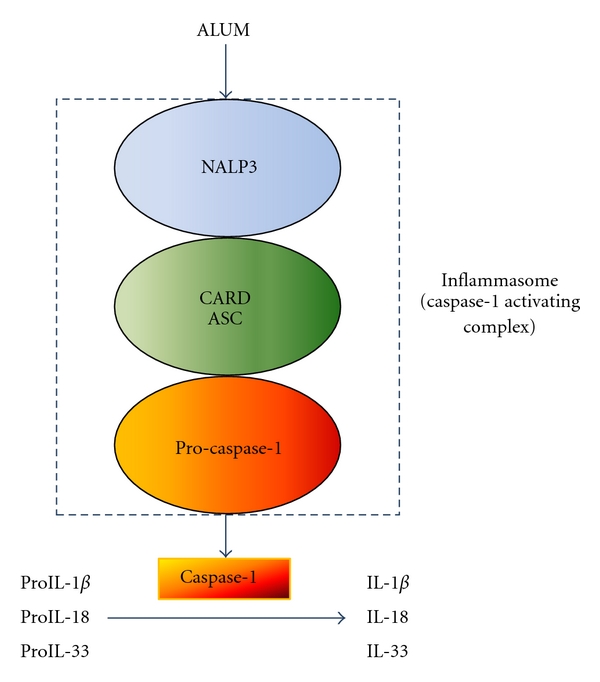
Scheme of the potential interaction of alum with the NALP3 inflammasome. Caspase-1 is activated, which in turn, promotes the activation of proinflammatory cytokines IL-*β*, IL-18, and IL-33. This process is abrogated by actin polymerization inhibitors, suggesting that activation of NALP3 requires phagocytosis.

Several action mechanisms have been proposed in order to explain alum adjuvancity. Previously, it was thought that alum formed a depot by which the antigen was slowly released and which converted the antigen into a particulate form, facilitating phagocytosis by APCs [35]. Later, it has been shown that alum induces inflammatory responses that recruit and activate APCs which capture the antigen [34]. Recent data demonstrate that alum targets NOD-like receptor protein 3 (NLRP3 or NALP3) to mediate caspase-1 activation and IL-1*β* release in lipopolysaccharide- (LPS-) primed macrophages [36]. NLRP3 interacts with Cardinal and ASC (apoptosis-associated speck-like protein) to form a caspase-1-activating complex called inflammasome, which, in turn, mediates the activation of proIL-1*β*, proIL-18, and proIL-33 into their active forms (Figure 2) [34]. However, *in vivo* data demonstrated that NLRP3 is dispensable for the adjuvant activity [36]. Nevertheless, other groups have reached conflicting conclusions. Eisenbarth et al. [37] and Li et al. [38] found an abrogation of the antibody responses to coadministered antigen in absence of NALP3 signaling, whereas Kool et al. [39] found only partial inhibition of the response. However, these results may be explained by the fact that different alum formulations were used in each study or different levels of TLR (Toll like receptor) agonist were used [40]. 

Other studies have suggested that NALP3 could be stimulated though indirect mechanisms. Kool et al. found that following alum administration, an increase in the endogenous danger signal uric acid happened. Neutralization of uric acid with uricase led to an inhibition of the inflammatory response induced by alum [33].

There are several investigators which study the immune response achieved after combining synthetic peptides with alum. For instance, a phase I clinical trial was conducted with the long synthetic peptide GLURP85-213 of *Plasmodium falciparum* combined with either alum or Montanide ISA as adjuvants [41]. Formulations were administered subcutaneously with 10, 30, or 100 *μ*g peptide doses at days 0, 30, and 120. Although serious adverse events were not observed, adverse events were more prevalent in the Montanide ISA group. On the other hand, both vaccines generated antibodies with capacity to mediate growth-inhibitory activity against *P. falciparum in vitro*.

However, nowadays, alum adjuvant is being replaced by other systems that improve the immune response achieved, and generally, it is used as a control or in combination with other adjuvants. For example, Raman et al. investigated the immunomodulatory effects of two types of CpG adjuvants intranasally administered with five synthetic peptide antigens of *Plasmodium vivax* in alum and microparticles. The addition of alum to CpG increased four-fold the antibody titers and triggered a predominance of IgG2a/2b isotypes. High titers against one of the peptides have a significant inhibitory effect on parasite development in the mosquito and the peptide-specific antisera reacted with the air-dried parasite antigens isolated from *P. vivax* patients [42].

### 2.2. Emulsions

Adjuvants composed of emulsions include oil in water (o/w) and water in oil (w/o) systems. There are two formulations approved for human use in Europe, MF59 and AS03. There is also another compound, Montanide, under phase III stage trials. 

MF59, a squalene-based o/w emulsion is licensed for influenza vaccine (Fluad). Vaccines with MF59 are safe and have demonstrated a better immunogenicity than nonadjuvanted ones, even in the elderly [44] and childhood [45]. Evaluation of safety data of 64 clinical trials involving MF59 revealed that MF59 adjuvanted subjects had lower risks than nonadjuvanted ones of undergoing unsolicited adverse events. On the other hand, MF59 adjuvanted subjects had a higher risk of expected local (mild or moderate pain, injection-site warmth induration, and erythema) or systemic reactions (myalgia, headache, fatigue, and malaise) [46]. The effects of the exposure to MF59 during pregnancy have also been evaluated. Tsai et al. analysed the clinical trial database of Novartis Vaccine studies from 1991 to 2009 and found that distribution of pregnancy outcomes (normal, abnormal, or ending in the therapeutic abortion) was similar in subjects exposed to MF59 compared to non exposed ones at any time of pregnancy, specifically in early pregnancy [47]. Although these data are few to draw definitive conclusions, available observations, so far, indicate no signal of risk.

Despite the wide use of MF59, its mechanism of action is not well understood. Immunofluorescence analysis showed that MF59 promoted antigen uptake by DCs after intramuscular injection [48], which suggest that its adjuvancity is not mediated by a depot effect. A study comparing the adjuvant effect of MF59, alum and CpG, characterized the changes in the expression of genes after intramuscular injection in mice. MF59 was the stronger inducer of cytokines, cytokine receptors, adhesion molecules involved in leukocyte migration, and antigen presentation genes [49]. In this study, it was hypothesised that MF59 combines the antigen delivery function with strong immunostimulating activity. Moreover, it may also promote a sustained antigen-presentation triggering the recruitment of CD11b^+^ monocytes, which might differentiate in functional inflammatory DCs, expressing high levels of MHC class II, as previously described for alum [33]. 

AS03 is a tocopherol o/w emulsion-based adjuvant used in Pandemrix, an influenza pandemic vaccine. Clinical trials have demonstrated that AS03 adjuvanted vaccines are able to trigger an immune response comparable to that obtained with nonadjuvanted ones using a fourfold lower dose [50]. In addition, the vaccine is well tolerated, and solicited adverse events are transient and mainly mild to moderate in intensity. Therefore, a high reduction in the dose of haemagglutinin can be achieved and can induce cross-clade immunity in humans, a prerequisite for an effective prepandemic vaccination strategy [51–53]. Moreover, a recent clinical trial suggests that Pandemrix used in children 6–35 months old is highly immunogenic and that overall reactogenicity profile is acceptable although reactions including fever tend to increase after a second dose [54]. However, to our knowledge, no study has been published that combines the use of synthetic peptides and MF59 or AS03.

Montanide is a w/o emulsion-based adjuvant. Although it is not yet approved for human use, lot of clinical trials are undergoing against several diseases such as malaria, melanoma, or nonsmall cell lung cancer [55]. A study carried out in our laboratory, compared the immune response against the S3 malarial synthetic peptide using Montanide, poly-lactide-co-glicolide (PLGA) microparticles and aluminium hydroxide. Subcutaneously administered Montanide and microspheres resulted in effective adjuvants and revealed mixed Th1/Th2 immune responses [56]. However, in a previous study it was shown that Montanide was effective in eliciting antibodies against the 3D7 peptide but not against the FC27 peptide [57]. In addition, a recent clinical trial has been carried out to evaluate the safety, tolerability, and immunogenicity of mixtures of N, R, and C long synthetic peptides derived from the *P. vivax* circumsporozoite protein formulated in two types of Montanide (ISA 720 and ISA 51) [58]. However, the results of this study are not yet published.

### 2.3. Polymeric Micro- and Nanoparticles

**Figure 3:**
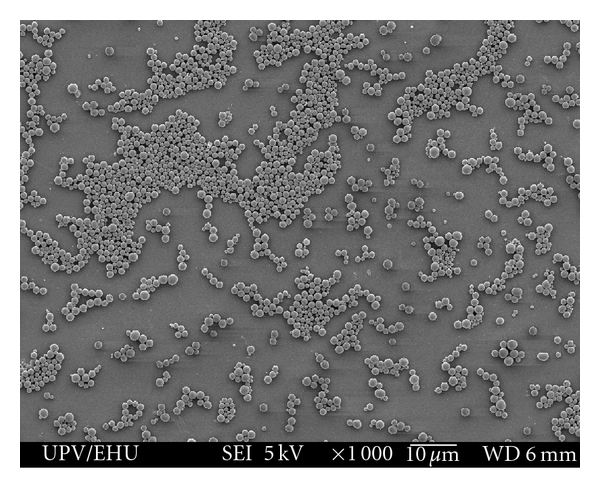
Scanning electron micrograph of PLGA microparticles (×10,000).

Polymeric micro- and nanoparticle-based vaccine delivery systems have been widely studied. The most commonly used polymers are poly(D,L-lactic-co-glycolic) acid (PLGA) and its derivates (Figure 3), due to their inherent advantages over other systems. They are biodegradable and biocompatible, are able to release molecules during long periods of time (weeks or months), and they are ease to administer via injection [59] or orally [60]. In addition, PLGA has been approved for human use in sutures [61], bone implants [62], and screws [63] as well as in implants for sustained drug delivery [64]. Apart from PLGA, other polymers have also been used for vaccination purposes, such as alginate [65], chitin [66], albumin [67], sodium polyacrylate [68], chitosan [69], poly-*ε*-caprolactone [70], or poly(*γ*-glutamic acid) [71] as well as some polymer combinations [72, 73].

In these formulations, the antigen can be either entrapped or adsorbed on the surface of the particles. The delivery of the antigen can be slow and continuous, by pulses or it can be triggered by external or environmental factors such as changes in the pH [74], temperature [75], ionic strength [76], or electric and magnetic fields [77].

The particle size and size distribution are important factors to determine antigen release rate, as the total surface area for protein delivery depends on the particle size [78]. With regard to particle size, it has been shown that it can influence the type of immune response achieved. In fact, nano- and microparticles (NPs and MPs) do not have the same behaviour *in vivo*. Kanchan and Panda showed that HBsAg-loaded polylactide MPs (2–8 *μ*m) elicited higher and long-lasting antibody titers and were not taken up by macrophages but were on their surface. In addition, MPs promoted IL-4 secretion and upregulation of MHC class II molecules and favoured Th2 immune response. On the other hand, NPs (200–600 nm) were efficiently phagocytized by macrophages and elicited lower antibody titers, but higher levels of IFN-*γ* production, upregulation of MHC class I molecules along with antibody isotypes favouring Th1-type immune response [79]. Moreover, Manolova et al. demonstrated that intradermally administered small-sized polystyrene particles (≤200 nm) were rapidly transported to the lymph nodes, where they were taken up by resident DCs. In contrast, large particles (500–2000 nm) depended on cellular transport by skin DCs [80]. Despite these differences, it is not clear which type of particle would be better for each particular case; therefore, particle size would be individually studied.

On the other hand, the administration route of particles may influence the immune response elicited. Mohanan et al. [81] have studied the bias of the immune response in mice when immunised by different routes, such as the subcutaneous, intradermal, intramuscular, and intralymphatic routes with ovalbumin-loaded liposomes, N-trimethyl-chitosan NPs and PLGA MPs, all with and without immune-response modifiers. This study has demonstrated that the IgG2a associated with Th1 immune response is sensitive to the route of administration, whereas IgG1 response associated with Th2 response was relatively insensitive to the administration route of particulate delivery systems.

Regarding to the mechanism of action, it has been shown that similarly to alum, PLGA microspheres enhance IL-1*β* secretion by DCs, in addition to trigger caspase-1 activation. These abilities require particle uptake by DCs and NALP3 activation [82]. Although the presence of a TLR agonist was required to induce IL-1*β* release *in vitro*, injection of the particles in the absence of a TLR agonist induced IL-1*β* production at the injection site, indicating that endogenous factors can synergize with particles to promote inflammasome activation. This study also showed that the enhancement of antigen-specific antibody production by microparticles was independent of NALP3, but it was needed in order to microspheres promote antigen-specific IL-6 production by T cells and recruitment and activation of CD11b^+^ Gr1^−^ cells. However, other studies showed that administration of LPS-modified PLGA microspheres loaded with antigen (ovalbumin), were preferentially internalized by DCs compared to nonmodified particles. In addition, these particles elicited potent humoral and cellular immunity against ovalbumin, and wild-type macrophages increased the release of IL-1*β*, consistent with inflammasome activation [83]. These data highlight that there is still controversy with the mechanism of action of polymeric micro- and nanoparticles.

PLGA micro- and nanospheres can be used for systemic or mucosal immunization [84–86]. PLGA-based systems are able to be phagocytosed by DCs, even by the oral route [87] and enhance their immunostimulatory capacity [88], leading to the upregulation of maturation markers CD40 and CD80 and release of IL-6. It has been shown that Hp91 synthetic peptide (a peptide that can induce potent antigen-specific cytotoxic T-lymphocyte responses), both encapsulated or conjugated to the surface of PLGA nanoparticles, is able to activate both human and mouse DCs more potently than the free peptide [88].

PLGA microspheres have been extensively studied by our research group. Different synthetic peptides have been entrapped into these microspheres, such as malarial SPf66, and have been administered by subcutaneous, intradermal [89], oral [17], or nasal [90] routes in mice. Microencapsulated SPf66 induced a superior immune response than the one obtained with the administration of the peptide adjuvanted with alum and comparable with the response obtained with FCA. In addition, these particles have been administered to Aoutus monkeys leading to high antibody levels and protection against *P. falciparum* challenge [91].

To our knowledge, only one clinical trial has been carried out using PLGA and synthetic peptides [92]. This phase I study evaluated the safety and immunogenicity of a synthetic HIV peptide (HIV-1 MN V3) administered intramuscularly with alum and a similar product encapsulated into PLGA microspheres administered by the oral route. However, the oral administration of this vaccine did not trigger significant humoral, cellular, or mucosal immune responses.

### 2.4. Liposomes

**Figure 4:**
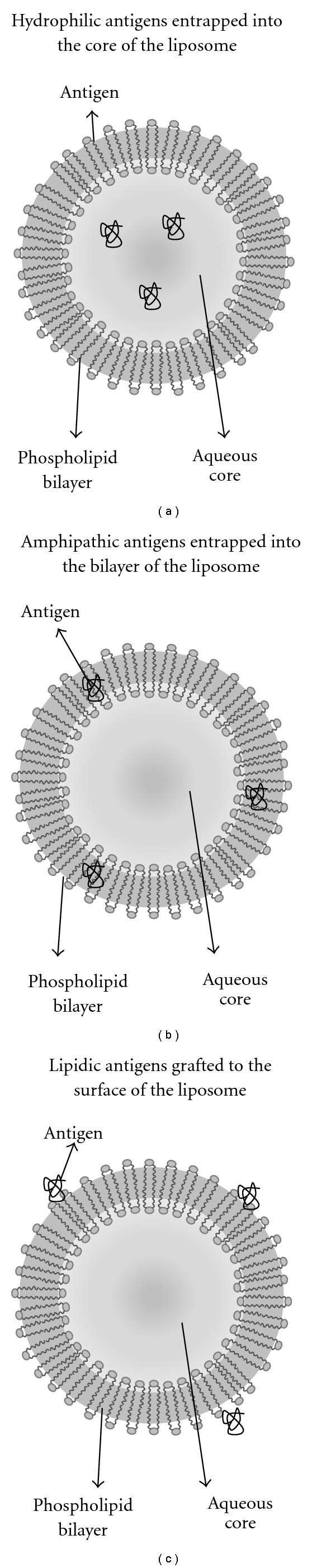
Scheme of liposomes structure. Antigens are differently incorporated based on their nature. Hydrophilic antigens can be encapsulated into the aqueous core; amphipathic antigens are integrated into the phospholipid bilayer, and lipidic antigens are adsorbed to the liposomes surface.

Liposomes are synthetic spheres comprised by phospholipid bilayers (Figure 4). According to their structure and size, liposomes can be classified into multilamellar vesicles (MLV), small unilamellar vesicles (SUV), intermediate unilamellar vesicles (IUV), or large unilamellar vesicles (LUV) [93]. For vaccine delivery, antigens can be encapsulated into the aqueous core, integrated in the lipid bilayer or adsorbed on the surface [4]. 

The mechanism of action of liposomes is not well defined. Passive targeting, derived of their particulate nature, and tendency to interact with macrophages is likely to be an important factor, particularly for nontargeted liposomes [94]. Among the different lipids available, cationic ones have a better ability to initiate and potentiate the immune response. It has been shown that positive charge is an important factor for the retention of liposomes at the injection site. Neutral liposomes have been shown limited in their ability to mediate long-term antigen presentation to circulating antigen-specific T cells and to induce the Th1 and Th2 arms of the immune system, as compared to cationic liposomes. The neutral liposomes did, however, induce the production of IL-5 at levels comparable to cationic liposomes, indicating that they can induce weak Th2 response [95]. 

Liposomes composition may also affect the type of immune response achieved. The inclusion of a fusogenic lipid in the formulation (i.e., easily fuses with the lipid membranes), such as DOPE, leads to superior IgG2a response against OVA, indicative of directing towards a Th1 response [96]. 

Coupling antigens to the liposomal surface can lead to CD4^+^, CD8^+^ T, and CTL immune responses. CTL epitopes composed of synthetic peptides derived from severe acute respiratory syndrome (SARS) coronavirus (SARS-CoV) and coupled to the surface of liposomes were effective for peptide-specific CTL induction in mice. One of these peptides was also able to clearance vaccinia virus which expresed epitopes of SARS-CoV after a challenge, suggesting that surface-linked liposomal peptides might offer an effective CTL-based vaccine against this disease [97]. On the other hand, it has been demonstrated that even small amounts of antigen entrapped into liposomes can induce IgG2a antibodies, the vias towards Th1 is more pronounced when more antigen is entrapped [96]. 

Liposomes can also induce antigen-specific antitumor immunity. Liposomes grafted to synthetic peptides derived from DCs maturation signals, such as HMGB1 (high-mobility group box 1), are able to target macrophages and DCs *in vitro* and *in vivo*. Coupling these liposomes to tumor derived plasma membrane vesicles inhibited tumor growth and metastasis after a tumor challenge in mice [98].

### 2.5. Virus Like Particles and Virosomes

**Figure 5:**
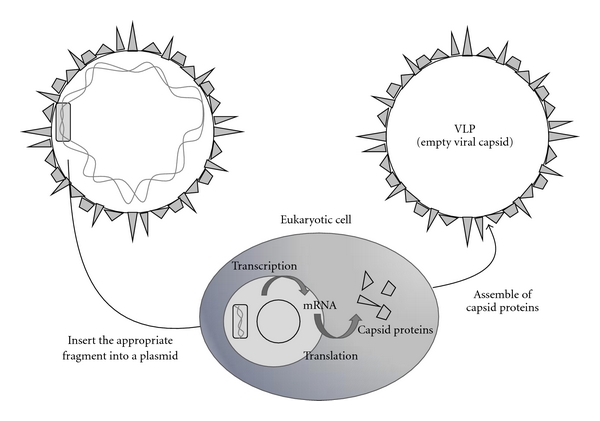
Preparation of VLPs. Viral genes encoding for the viral capsid proteins are inserted into a plasmid, which is transcripted and translated in a eukaryotic cell. Viral capsid proteins are synthetised and assemble spontaneously into VLPs.

Virus like particles (VLPs) are obtained when viral structural proteins are produced in recombinant expression systems or even in cell-free systems [99, 100]. Recombinant viral structural proteins of several viruses can spontaneously assemble into VLPs in the absence of the viral genetic material and other viral proteins, which makes them non infectious (Figure 5). VLPs are able to incorporate peptide vaccines, either produced by recombination (genetically fused to the gene which encodes for the VLP), or chemically coupling peptides to the formed VLP [101, 102]. Pejawar-Gaddy et al. generated bovine papillomavirus (BPV) VLPs that were chemically coupled to a synthetic derivate of MUC1 (human mucin-1) peptide [103]. This peptide is aberrantly expressed on a wide range of ductal adenocarcinomas and has been intensively studied as a candidate cancer vaccine antigen. MUC1-conjugated VLPs were subcutaneously administered to MUC1 transgenic mice, leading to a robust activation of bone marrow-derived DCs, which presented the antigen to MUC1-specific T cells. In addition, immunization of human MUC1 transgenic mice, where MUC1 is a selfantigen, with the VLP vaccine induced MUC1-specific CTL, delayed the growth of MUC1 transplanted tumors and elicited complete tumor rejection in some animals. This study and others [102, 104] demonstrate that VLP could be efficiently taken up by APCs, leading to both MHC class II and I presentation. In addition, VLPs are able to induce potent antivirus humoral and cellular immune responses [105–107]. 

Several vaccines based on VLPs are currently approved for human use (Gardasil and Cervarix), demonstrating that VLP provide an appropriate immunity against papillomavirus [27, 29, 108]. Moreover, other VLP-based vaccines are under development, including vaccines against influenza [109, 110], HIV [111], or Norwalk virus [112], and in clinical trials [113].

**Figure 6:**
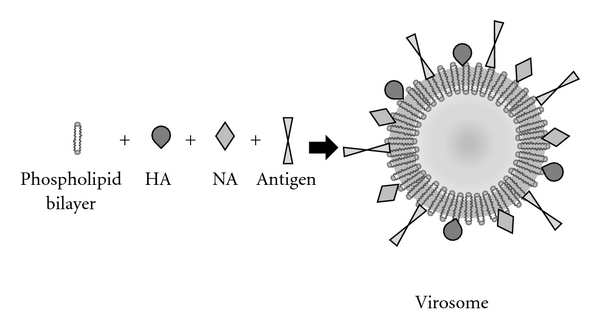
Composition of a virosome derived from influenza virus. The envelope of the virus is composed of a phospholipid bilayer and is similar to a liposome. This structure is used as a platform to which other viral components are incorporated. Influenza viruses are often used to prepare virosomes, which maintain the properties of viral haemagglutinin (HA) and neuraminidase (NA). Furthermore, other antigens can be incorporated into the system, allowing the vaccination against other microorganisms.

Virosomes are similar to virus-like particles, consisting of reconstituted viral envelopes lacking the viral genetic material. They are generated from virus by a detergent solubilization and reconstitution procedure [114]. The main difference with VLPs is that VLPs are self-assembled viral capside proteins, while virosomes use the envelope phospholipid bilayers as a platform to which additional viral components or antigens are attached (Figure 6) [4]. Virosomes may be produced from a variety of enveloped viruses although the most used one is the influenza virus. In fact, virosomal approved vaccines (Inflexal and Epaxal) are composed of influenza virosomes [24, 25]. Influenza virosomes possess membrane fusion properties very similar to the native virus, because they maintain the receptor-binding and membrane fusion activity of the viral haemagglutinin. Therefore, virosomes enter cells through receptor-mediated endocytosis, but this process does not result in the infection of cells, because virosomes lack the viral RNA [115].

Foreign macromolecules, including synthetic protein antigens, can be encapsulated in virosomes during the reconstitution process. These virosomes are able to induce a powerful class I MHC-restricted CTL response, mainly because they will deliver their content to the cell cytosol [116], which favours the cross-presentation. This makes virosomes possible to be used as a suitable delivery system in tumor immunotherapy [117].

On the other hand, a fraction of the particles will inevitably be degraded within the endosomal/lysosomal compartment. The resulting peptides will be able to associate with MHC II molecules, resulting in CD4^+^ response [116]. Development of antibody responses have been found upon administration of malarial synthetic antigens containing virosomes. In fact, IgG antibodies against UK-39 (a synthetic peptide derived from the circumsporozoite protein of *P. faciparum*) inhibited invasion of hepatocytes by *P. falciparum* sporozoites [118]. A second peptide (AMA49-C1) based on domain III of apical membrane antigen 1 induced antibodies that inhibited blood-stage parasite growth *in vitro* [119]. Combination of both antigens into different virosomes did not affect negatively the antipeptide antibody titers in mice or rabbits, demonstrating the value of this system for the development of multivalent vaccines [120]. In addition, a phase I clinical trial has been carried out in order to evaluate the safety and immunogenicity of two virosome-formulated *P. falciparum* derived synthetic peptide antigens (AMA 49-CPE and UK39) [121]. Both vaccines resulted safe, as no serious or severe adverse events were observed. In terms of immunogenicity, both formulations elicited already an antibody specific response in all volunteers with the appropriate dose.

### 2.6. ISCOMS and ISCOMATRIX

**Figure 7:**
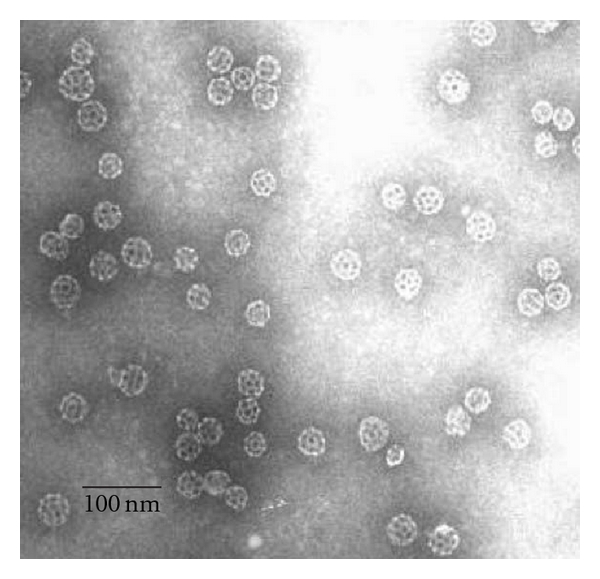
Electron micrograph of ISCOMATRIX adjuvant following negative staining. ISCOMATRIX adjuvant particles are typically rigid, hollow, spherical, and cage-like particles approximately 40 nm in diameter. Reproduced with permission from [43].

Immunostimulatory complexes (ISCOMs) are cage-like structures, approximately of 40 nm in diameter composed of antigen, cholesterol, phospholipid, and saponin, held together by hydrophobic interactions, so typically entrapped antigens are amphipathic. The most commonly used saponin is QuilA or its purified compounds [5, 122]. ISCOMATRIX has essentially the same structure as ISCOMs but lacks the antigen, which can be subsequently added (Figure 7). This fact provides ISCOMATIX for more general applications as they are not limited to amphipathic antigens [4, 122]. Although numerous studies have been carried out with animal models [123–126], few clinical trials evaluating ISCOMs and ISCOMATRIX are currently in course [127].

ISCOMs are not immunogenic by themselves although other saponins different from QuilA are used [43, 128], but when the antigen is incorporated, they can trigger humoral, mucosal, and cellular immune responses [128]. Different results have been obtained when evaluating ISCOMs immunogenicity. For instance, Agrawal et al. [129] administered in the footpad of mice different HIV-1 derived synthetic peptides, with and without an immunoadjuvant, in liposomes or ISCOMs and compared to the administration of peptides with alum. In contrast to alum, both liposomes and ISCOMs induced a predominant Th1 like response. On the other hand, Pahar et al. [123] found that intrarectal immunization of macaques with two HIV-derived peptides (HIV-1_env_ and SIV_gag_) incorporated into ISCOMs induced low level of immunity against simian-HIV. These differences may be due to the antigens used, differences in the administration route, dose, or schedule.

ISCOMATRIX adjuvant facilitates antigen delivery and presentation as well as immunomodulation to provide enhanced and accelerated immune responses. Moreover, it is capable of inducing broad and potent humoral and cellular immune responses including both CD4^+^ and CD8^+^ T cell responses [130, 131]. The antibody response is often achieved with lower amounts of antigen than with other adjuvant systems [132]. Additionally, ISCOMATRIX adjuvant can be used in vaccines for induction of mucosal immune responses [133, 134]. In fact, protective ability of ISCOMATRIX adjuvanted vaccines has been reported [135], and they have been used in some veterinarian vaccines [136]. 

ISCOMATRIX adjuvants are also effective in the field of cancer treatment. NY-ESO-1 is a protein expressed in many cancers. This recombinant protein with ISCOMATRIX adjuvant has been evaluated in a clinical trial [137] demonstrating that the vaccine is safe and highly immunogenic. Recently, Ebert et al. have studied the effects of a NY-ESO-1 peptide synthetic derivate (NY-ESO-1_60-72_/HLA-B7 tetramer) with ISCOMATRIX in humans. They have found that this vaccine formulation allows DCs to cross-present the NY-ESO-1_60-72_ epitope efficiently and generates a potent T cell response.

Regarding to safety concerns, Anderson et al. have pooled and analyzed the safety data obtained from a number of vaccine development programs comprising ISCOMATRIX. Overall, the ISCOMATRIX vaccines were found to be safe and well tolerated, with no vaccine-related deaths or serious adverse events. Reactogenicity at the injection site was found to be the most frequent adverse event compared with subjects who received placebo or active comparator; however, this reactogenicity was generally mild, self-limiting, and of short duration. Until the end of the study, ISCOMATRIX vaccines have not been associated with events suggestive of autoimmune or allergic disorders nor events of anaphylaxis [138]. 

Recently, cationic immune stimulating complexes have been developed (PLUSCOMs). In contrast to ISCOMs, PLUSCOMs are able to incorporate hydrophilic peptides adsorbed onto their surfaces by ionic interactions. In addition, they are as effective as classic ISCOMs in inducing antigen-specific CD8^+^ T cell responses [139].

### 2.7. Nanobeads

The use of nanobeads as vaccine carrier/adjuvant systems implies the coupling of solid inert beads, generally made of carboxylated polystyrene, with an antigen [5]. Beads of 40–50 nm are better internalized by DCs than higher ones and induce CD8^+^ type immune response, whereas larger beads facilitate CD4^+^ response [140]. Other studies carried out *in vivo* were in accordance to this finding. Particles in this size range could elicit antibody and cell immunity in mice, as well as provide protection after a tumor challenge [9, 141]. Later, these findings were also confirmed in sheep [142, 143]. For instance, administration of multiple synthetic peptides derived from foot-and-mouth disease virus conjugated separately to individual nanobeads or conjugated as a mixture, were able to induce significant cell-mediated and humoral immune responses in sheep administered intradermally [143].

## 3. Current Approaches to Improve the Immunogenicity of Particulated Systems

The development of successful vaccines implies the production of an appropriate immune response against a given pathogen. This approach concerns immunological, biotechnological, and pharmaceutical aspects, as the interaction between DCs and T lymphocytes, selection of appropriate antigens and adjuvants, and the production of an stable end product must be taken into account [15]. In some cases, vaccine delivery systems have been sufficient to induce a long lasting protective immunity. However, poorly immunogenic antigens, such as synthetic peptides, are often unable to induce a protective immunity when incorporated into delivery systems alone and require the incorporation of immune potentiating molecules [8]. Immune potentiators activate innate immune receptors of APCs (named pathogen recognition receptors—PRRs), which recognize pathogen associated molecular patterns (PAMPs). Among PRRs, signalling receptors act as primary sensor of pathogens and damage, and finally trigger both effector and adaptive immune responses. These receptors can be located on the plasma membrane, in different internal compartments, or in membranes from intracellular vesicles, or can be cytosolic proteins [144]. Three families of signalling receptors have been identified: TLRs, NLRs, and RLRs. Members of TLR family recognize bacteria, viruses, fungi and protozoa; NLRs detect bacteria and RLRs are antiviral. It is likely that interaction between these families provides synergistic or cooperative signalling [145]. In addition, other PPRs (humoral proteins and endocytic receptors) have a critical role in activating antigen presentation [144, 146].

The activation of PRRs by immune potentiators induces the secretion of proinflammatory cytokines and type I interferon, the upregulation of costimulatory molecules and MHC class II molecules. In addition, PRRs also trigger the migration of APCs from the injection site to the T cell areas of the draining lymph nodes. All these processes are needed for activation of naive T cells and the development of both humoral and cellular immune responses [147]. Thus, formulation of vaccines that target PRRs is an interesting approach in order to improve their immune response. 

Traditionally, antigens have been formulated into their soluble form plus an immune potentiating molecule [148, 149] or were entrapped into delivery systems alone [89, 150]. Current tendency is to combine more than one adjuvant into the same vaccine formulation in order to achieve the desired immune response.

### 3.1. Combination of Adjuvants with Different Action Mechanism

It has been shown that combination of adjuvants can increase the immune response. For this reason, most of the novel vaccine formulations comprise a combination of adjuvants.

The most common combination of adjuvants with different action mechanism is the use of a vaccine delivery system which contains the antigen and an immune potentiating adjuvant. For instance, combination of PLGA nanoparticles coencapsulating the poorly immunogenic melanoma antigen, tyrosinase-related protein 2 (TRP2), along with Toll-like receptor 4 ligand (TLR4) (7-acyl lipid A) led to a therapeutic antitumor effect against melanoma after the subcutaneous administration to mice [151]. 

Although they do not contain synthetic peptides, some of the licensed vaccines are comprised of a mixture of adjuvants, such as Gardasil (composed of VLPs and alum), Fendrix (comprising AS04, approved for human use in Europe and USA), or Cervarix (which includes AS04 and VLPs). These vaccines present a high immunogenicity and are safe [26, 152].

### 3.2. Targeting Antigens to Dendritic Cells

Vaccine delivery systems can incorporate ligands in order to specifically target APCs receptors. It has been shown that ligand grafting can enhance the uptake of microparticles by immune cells. Brandhonneur et al. [153] studied the uptake of different ligand-grafted PLGA microspheres by alveolar macrophages of pigs *ex vivo*. Three different ligands were used: WGA (lectin weat germ agglutinin, which interacts with lectin receptors), a RGD (arginine-glycine-aspartate) containing peptide (interacting on integrins), and a carbohydrate moiety (manose-PEG_3_-NH_2_, interacting on manose receptor). A much higher uptake was observed for mannose-, WGA-, and RGD-grafted microspheres, mainly because of the specific mechanism of phagocytosis. When other ligands were grafted to the microspheres (peptides like BSA—bovine serum albumin or RAD—arginine-alanine-aspartame), the uptake was not significantly different from ungrafted microspheres, due to the nonspecific mechanism of uptake, given the lack of receptors for BSA and RAD into macrophages.

Among PRR ligands, TLR ligands have been widely studied. TLR activation leads to upregulation of CD40, CD80, CD86, and CD70 costimulatory molecules in the surface of APCs, as well as release of Th1 cytokines such as IL-1, IL-2, IL-6, and TNF. In addition, some ligands are able to trigger cross-presentation. Therefore, TLRs facilitate coordination between innate and adaptive immunities by activating B and T cells as well as memory responses [154]. It has been shown that antigens and TLR ligands can generate more potent immune responses when coencapsulated into the same particle [155]. This can be understood taking into account that endosomal organelles of DCs express some TLRs, in addition to posses machinery to process captured antigens and present them into MHC molecules. Consequently, simultaneous delivery of antigen and TLR-ligands into the cytosol may lead to a better DC activation and subsequent development of immune response.

There exist at least 13 members of TLRs, which recognize different microbial components. For instance, TLR2 recognize bacterial lipoproteins and lipopeptides in cooperation with TLR1 or TLR6 [156], TLR4 binds LPS [157], TLR3 recognizes double stranded RNA [158], TLR5 attaches to flagellin [159], TLR7 and TLR8 recognize single-stranded viral RNA [160] and synthetic imidazoquinolines [161], and TLR9 recognizes DNA rich in nonmethylated CpG (cytosine-phosphorothioate-guanine) [162]. 

One of the most widely used immunopotentiating adjuvants are those which interact with TLR9, either CpGs present into bacterial or viral DNA or synthetic CpG oligodeoxynucleotides (CpG ODN) [163]. Vaccination with liposomes containing synthetic peptides derived from lymphocytic choriomeningitis virus (LCMV) and CpG motifs by intramuscular route, resulted in the efficient induction of antiviral CD8^+^ T cell responses and complete protection against not only LCMV but also against a highly virulent mutant strain. Moreover, the intranasal administration induced mucosal immunity able to protect mice from the virus challenge, even using a low dose [164].

Other frequently used TLR ligands are those directed to TLR3. Poly(inosinic-cytidilic) acid, that is, poly(I:C), is a synthetic analogue of double-stranded RNA which exerts its function via TLR3 [165]. Poly(I:C) induces maturation of DCs [166], is a potent IFN inducer and can activate monocytes and NK cells to produce proinflammatory cytokines and chemokines [167]. Furthermore, poly(I:C) is able to enhance specific antitumor immunity against synthetic peptide-based vaccines by inducing CTL response [168], mainly because it allows cross-priming [169]. It has been shown that fluorescent-BSA-loaded PLGA microparticles including poly(I:C) are effectively phagocytized by DCs *ex vivo* and induce a maturation similar to that achieved with a cytokine cocktail or higher concentrations of soluble poly(I:C) [170]. Besides, murine splenic DCs pulsed with polyketal-OVA-poly(I:C) microparticles induce higher percentage of IFN-*γ*-producing CD8^+^ T cells than DCs treated with polyketal-OVA particles or soluble OVA/poly(I:C) [171].

In addition to targeting TLRs, other delivery systems have been prepared which target other DC receptors. These carriers incorporate antibodies or molecules that specifically interact with receptors such as DC-SIGN [172] or DEC-205 [173] and have the ability to trigger the phagocytosis of entrapping synthetic peptides by DCs and promote their maturation.

## 4. Conclusion

Vaccination with subunit vaccines comprised of synthetic proteins and peptides is not always successful, because they can be degraded by proteases, possess limited bioavailability, and present relatively low immunogenicity. Delivery systems are able to overcome these problems, since they protect proteins from degradation and increase their bioavailability allowing the cross of biological membranes. With regard to immune response, delivery systems can improve and/or modulate the response achieved with soluble peptides alone. Although it has been proposed that they exert their adjuvancity by generating a depot effect at the injection site, currently, other action mechanism have been found which better explain the modulation or improvement of the immune response. Carriers can be passively directed and subsequently endocyted by APCs and deliver the antigen to the cytosol or intracellular organelles. In addition, they can interact with protein complexes, such as inflammasome, to activate immune response. Furthermore, they can incorporate other immunostimulatory molecules which may improve or modulate the immune response in order to develop not only humoral but also cellular immunity.

Delivery systems also possess other advantages; they are safe, stable, and reproducible. Besides, they can be administered by several routes, which offer the possibility of developing both mucosal and systemic immune responses.

All these features have led to the approval of some of these systems to clinical use, such as VLPs, virosomes, or traditional alum. Although these adjuvants are able to trigger appropriate immune responses against certain pathogens, the future in this field will be focused on the development of combined vaccines to better design the induction of an appropriate immune response.

## Figures and Tables

**Table 1:** Examples of EMA- and/or FDA-approved vaccines based on micro- and nanoparticulated delivery systems. MF59 and AS03 are squalene- and tocopherol-based o/w emulsions, respectively. AS04 is composed of monophosphoril lipid A and alum. Virosomes are composed of viral-derived phospholipid bilayers, and virus-like particles (VLPs) are viral capsids lacking genetic material.

Vaccine	Delivery system	Indication	Reference
Recombivax	Alum	Hepatitis B	[17]
Engerix B	Alum	Hepatitis B	[18, 19]
Tripedia, Infanrix, DAPTACEL	Alum	Diphteria, Tetanus and Pertussis	[16]
Fluad	MF59	Influenza/pandemic flu	[20]
Pandemrix	AS03	Pandemic flu	[21]
Fendrix	AS04	Hepatitis A	[24]
Epaxal	Virosomes	Hepatitis A	[22]
Inflexal	Virosomes	Influenza	[23]
Cervarix	AS04 + VLPs	Human papillomavirus	[25, 26]
Gardasil	VLPs + Alum	Human papillomavirus	[27]

**Table 2:** Schematic view of the mechanism of action and advantages of the different micro- and nanotechnologies for peptide-based vaccine delivery.

Type of technology	Role	Advantages
Alum	(i) Depot (ii) Activation of inflamasome and IL-1*β* release	(i) Enhances antibody responses

Emulsions	(i) Promote antigen uptake by DCs (ii) Strong immunostimulatory activity	(i) Allows reduction of antigen dose
		(ii) Well tolerated
		(iii) Useful in children
		(iv) Mixed Th1/Th2 responses

Polymeric MPs and NPs	(i) Enhance IL-1*β* secretion by DCs	(i) Biodegradable and biocompatible
		(ii) Release during long time periods
		(iii) Modulation of the delivery: continuous, by pulses, or triggered by several factors (pH, temperature, ionic strength, electric or magnetic fields)
		(iv) Elicit humoral and cellular immunity

Liposomes	(i) Passive targeting (ii) Tendency to interact with macrophages	(i) CD4^+^, CD8^+^ and CLT immune responses
		(ii) Modulation of the immune response using different lipids

VLPs	(i) Taken up by APCs and MHC class I and II presentation	(i) Incorporation of peptides produced by recombination, or chemically coupling them once the VLP is formed
		(ii) Potent humoral and cellular immune responses

Virosomes	(i) Enter cells through receptor mediated endocytosis	(i) Membrane fusion properties of the virus are maintained
		(ii) Humoral and CTL responses
		(iii) Value for developing multivalent vaccines

ICOMs and ISCOMATRIX	(i) Antigen carrier (ii) Immunostimulation (because of the saponin)	(i) Potent humoral and cellular immune responses
		(ii) Reduction of the antigen dose
		(iii) Safe and well tolerated

Nanobeads	(i) Depends on the size: small ones elicit CD8^+^ immune response, whereas larger ones facilitate CD4^+^ responses	(i) Humoral and cellular immune responses

## Abbreviations

APCs: Antigen-presenting cells
ASC: Apoptosis-associated speck-like *protein *
BCG: Bacillus Calmette-Guerin
BPV: Bovine papillomavirus
BSA: Bovine serum albumin
CoV: Coronavirus
CpG: Cytosine-phosphorothioate-guanine
CTL: Cytolytic immune response
DCs: Dendritic cells
HBsAg: Hepatitis B surface antigen
HIV: Human immunodeficiency virus
HMGB1: High-mobility group box 1
IFN: Interferon
IL: Interleukin
IP: Inducible protein
ISCOMs: Immunostimulatory complexes
IUV: Intermediate unilamellar vesicles
LCMV: Lymphocytic choriomeningitis virus
LUV: Large unilamellar vesicles
MHC: Major histocompatibility complex
MLV: Multilamellar vesicles
MPL: Monophosphoril lipid A
MPs: Microparticles
MUC1: Human mucin-1
NKT cells: Natural killer T cells
NLR: Nod-like receptor
NLRP3 or NALP3: NOD-like receptor protein 3
NPs: Nanoparticles
o/w: Oil in water
OVA: Ovalbumin
PAMPs: Pathogen associated molecular patterns
PLGA: Poly(D,L-lactic-co-glycolic) acid
PLUSCOMs: Cationic immune stimulating complexes
Poly(I:C): Poly(inosinic-cytidilic) acid
PRRs: Pathogen recognition receptors
RAD: arginine-alanine-aspartame
RGD: arginine-glycine-aspartate
RLR: Rig-like receptor
RNA: Ribonucleic acid
SARS: Severe acute respiratory syndrome
SUV: Small unilamellar vesicles
TLR: Toll like receptor
TRP2: Tyrosinase-related protein 2
VLPs: Virus like particles
w/o: Water in oil
WGA: Lectin weat germ agglutinin.
